# Transfer function analysis assesses resting cerebral perfusion metrics using hypoxia-induced deoxyhemoglobin as a contrast agent

**DOI:** 10.3389/fphys.2023.1167857

**Published:** 2023-05-03

**Authors:** Ece Su Sayin, Olivia Sobczyk, Julien Poublanc, David J. Mikulis, Joseph A. Fisher, James Duffin

**Affiliations:** ^1^ Department of Physiology, University of Toronto, Toronto, ON, Canada; ^2^ Departments of Anaesthesia and Pain Management, University Health Network, Toronto, ON, Canada; ^3^ Joint Department of Medical Imaging and the Functional Neuroimaging Laboratory, University Health Network, Toronto, ON, Canada; ^4^ Toronto General Hospital Research Institute, University Health Network, University of Toronto, Toronto, ON, Canada

**Keywords:** transfer function analysis, transient hypoxia, MRI, BOLD = blood oxygen level dependent, contrast agents, brain, perfusion imaging

## Abstract

**Introduction:** Use of contrast in determining hemodynamic measures requires the deconvolution of an arterial input function (AIF) selected over a voxel in the middle cerebral artery to calculate voxel wise perfusion metrics. Transfer function analysis (TFA) offers an alternative analytic approach that does not require identifying an AIF. We hypothesised that TFA metrics Gain, Lag, and their ratio, Gain/Lag, correspond to conventional AIF resting perfusion metrics relative cerebral blood volume (rCBV), mean transit time (MTT) and relative cerebral blood flow (rCBF), respectively.

**Methods:** 24 healthy participants (17 M) and 1 patient with steno-occlusive disease were recruited. We used non-invasive transient hypoxia-induced deoxyhemoglobin as an MRI contrast. TFA and conventional AIF analyses were used to calculate averages of whole brain and smaller regions of interest.

**Results:** Maps of these average metrics had colour scales adjusted to enhance contrast and identify areas of high congruence. Regional gray matter/white matter (GM/WM) ratios for MTT and Lag, rCBF and Gain/Lag, and rCBV and Gain were compared. The GM/WM ratios were greater for TFA metrics compared to those from AIF analysis indicating an improved regional discrimination.

**Discussion:** Resting perfusion measures generated by The BOLD analysis resulting from a transient hypoxia induced variations in deoxyhemoglobin analyzed by TFA are congruent with those analyzed by conventional AIF analysis.

## 1 Introduction

Cerebral blood flow is distributed via a complex network of vessels with flow resistances that vary depending on anatomy, vascular health, and tissue metabolism. This distribution of blood flow can be mapped using blood oxygenation level dependent (BOLD) magnetic resonance imaging (MRI) to trace a bolus of contrast agent. The BOLD signal is sensitive to distortions in the static magnetic field caused by paramagnetic contrast agents such as gadolinium (Ogawa et al., 1990). The value of resting perfusion metrics obtained from the passage of a contrast agent during BOLD imaging, including mean transit time (MTT), relative cerebral blood volume (rCBV) and relative cerebral blood flow (rCBF), is that they can indicate regions of slowed and insufficient resting blood supply (Donahue et al., 2017). The dynamic susceptibility contrast (DSC) agent of choice used for perfusion imaging clinically is a gadolinium-based contrast agent (GBCA), injected intravenously to generate a bolus that is required to be imaged over a large artery on its first pass through the brain. Tissue BOLD signals can be analysed during passage of the GBCA bolus through the brain using the first pass arterial signal changes (arterial input function or AIF) deconvolved with tissue signal changes to obtain tissue perfusion metrics.

Transient hypoxia-induced deoxyhemoglobin (THx-dOHb) can be used as an endogenous paramagnetic contrast agent for DSC imaging. In 2021 Poublanc et al. reported the use of THx-dOHb as a non-invasive dynamic susceptibility contrast agent (Poublanc et al., 2021; Vu et al., 2021; Sayin et al., 2022a). Comparisons of resting perfusion metrics calculated from THx-dOHb were very similar to those obtained from a clinical standard, GBCA (Sayin et al., 2022a). However, a significant issue with DSC perfusion mapping is that the BOLD signal in proximity to arteries has both linear and non-linear behaviour depending on where the signal is measured. The intravascular signal exhibits a quadratic relationship with the concentrating of the paramagnetic contrast agent (Spees et al., 2001; Zhao et al., 2007) at 3 Tesla (Uludag et al., 2009), whereas the signal in tissue adjacent to the vessel is linear with contrast concentration. The nature of the AIF used for perfusion analysis is therefore dependent on location of the voxels used to measure the AIF. An AIF independent method for acquiring DSC perfusion metrics would therefore be welcomed.

### 1.1 Transfer function analysis

**FIGURE 1:**
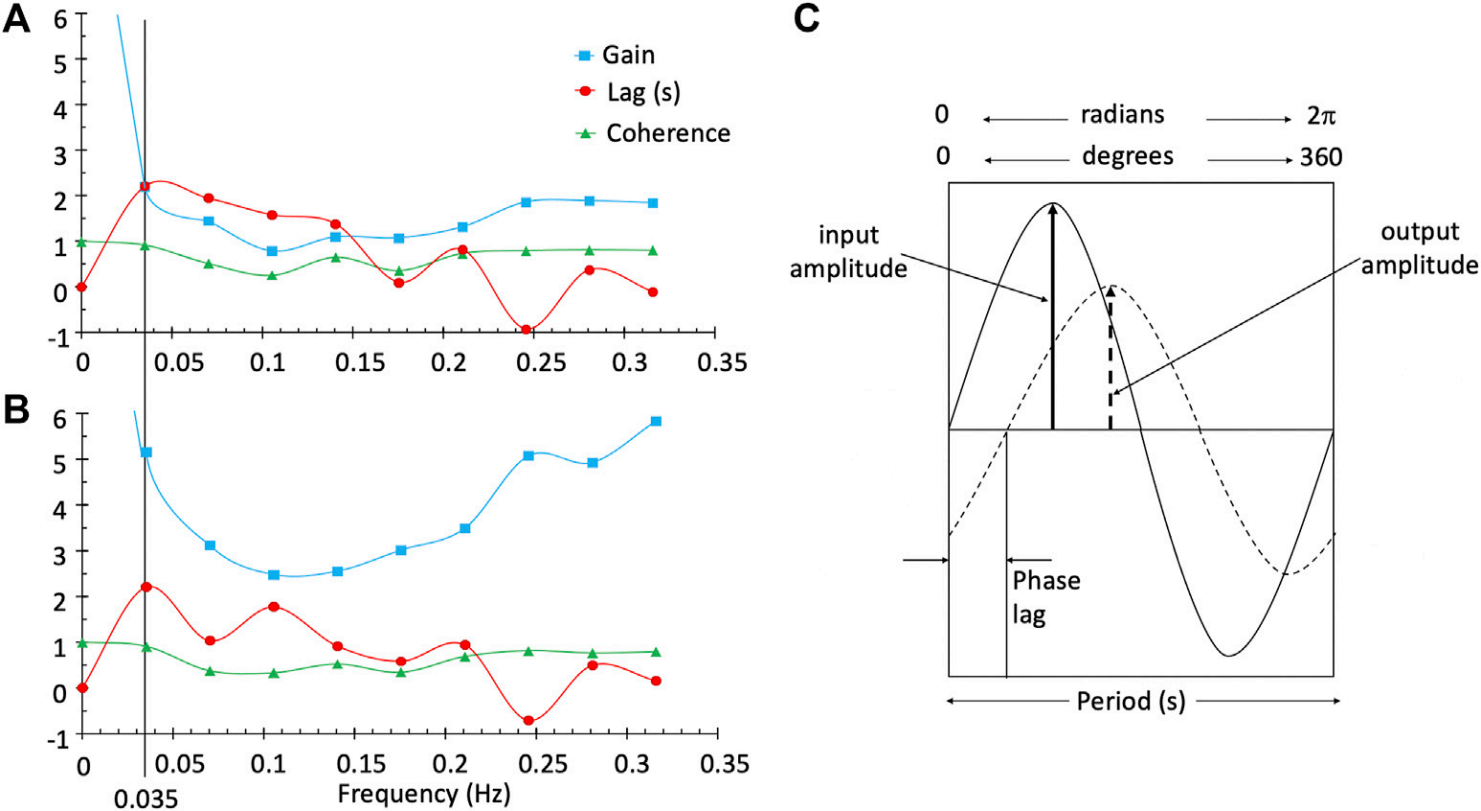
TFA provides the Gain, phase or time Lag, and Coherence measures for all frequencies in a single voxel in the white matter **(A)** and a single voxel in the grey matter **(B)**. The line at a frequency of 0.035 Hz was chosen as the single frequency representative measurement of Gain, Lag and Coherence used to characterise the BOLD response in a voxel. Diagram **(C)** illustrates a single frequency. Gain is output amplitude/input amplitude and phase is the difference in the timing of the sine waves. As illustrated, the output is positive and lags the input; consequently, phase is negative. The time lag (s) is period*phase lag (r/2π).

Here we introduce a frequency domain analysis technique, transfer function analysis (TFA). This technique has been used to measure dynamic pressure autoregulation of the cerebral vasculature (Blaber et al., 1997; Zhang et al., 1998; Tzeng et al., 2012), as well as the cerebrovascular response to changes in CO_2_ (Duffin et al., 2015). It offers a means of characterizing the BOLD response to [dOHb] with not only an estimate of the magnitude of the response (Gain), but also the phase, or time Lag (Blockley et al., 2011) and coherence, the linear time invariance of the BOLD to SO_2_ relationship. Briefly, the aligned BOLD response and SO_2_ data are divided into five 50% overlapping segments (Welch algorithm (Welch, 1967)). In each segment the relation between the BOLD response signal and the contrast signal (SO_2_) is analyzed in the frequency domain by resolving the two signals into their Fourier series of component sine waves (Figures 1A, B). The frequency response function, defined as the average cross-spectrum of the response signal divided by the average autospectrum of the stimulus signal, yields Gain and Phase measures, averaged for all segments. Coherence is calculated from averages of the cross- and auto-spectra as the average cross-spectrum squared divided by the product of the stimulus and response autospectra. Gain, Phase and Coherence measures are taken from the frequency spectrum at a single reference frequency (Figures 1A, B). As Figure 1C illustrates, Gain describes the amplitude ratio relating the BOLD response to the contrast signal and phase or time Lag the time relationship.

Previously, TFA was used to describe the dynamics of the BOLD response to changes in a vasoactive agent such as CO_2_ (Duffin et al., 2015; Sayin et al., 2022b). In this case the phase difference between stimulus and response arises from two factors: a blood arrival time delay and a vascular response time. Differences in time delay between regions were assumed to be less than the 1.5 s sampling period (TR) so that TFA phase primarily reflects the speed of the vascular response (Duffin et al., 2015). By contrast, the phase difference between [dOHb] changes and the resulting BOLD signal is assumed to arise from both the time of passage of blood through a voxel and the arrival time delay. If the time of arrival delay is minimised, the phase difference expressed in time units is a measure related to mean transit time (MTT). We further hypothesised that if the Gain can be assumed to reflect the strength of the signal in a voxel, which is proportional to the volume of blood in a voxel, then Gain is a measure of relative cerebral volume (rCBV). In this case the ratio of Gain/Lag (CBV/MTT) is a measure of relative cerebral blood flow (rCBF), analogous to the central volume theorem (Ostergaard, 2005).

### 1.2 Observations

We describe the utility of transfer function analysis of the BOLD response to a series of two transient hypoxic exposures to produce corresponding changes in [dOHb] at rest. Voxel-wise maps of Gain, Lag and Gain/Lag were assembled by averaging these TFA metrics calculated and compared with maps of rCBV, MTT and rCBF respectively obtained from a deconvolution-based method using an AIF as described by Poublanc et al. (2021). Average supratentorial whole brain and posterior/anterior circulation TFA perfusion metrics were also compared to the corresponding conventional AIF analysis. In addition, we made spatial comparisons of the maps in a healthy participant and a patient with vascular insufficiency as shown in the magnetic resonance angiography (MRA) in Figure 8.

## 2 Materials and methods

### 2.1 Participant and ethics approval

This study conformed to the standards set by the latest revision of the Declaration of Helsinki and was approved by the Research Ethics Board of the University Health Network (UHN) and Health Canada. All participants provided written and informed consent to partake in this study. We recruited 24 healthy participants (7 Female) ranging from 21 to 82 (mean (SD) 38.6 ± 17.9 years, median age 30 years (Table 1) by word of mouth. The healthy participants consisted of non-smokers, not taking any medication and no known history of neurological or cardiovascular disease. In addition, a 66-year-old female participant with known cerebral vascular disease was recruited. The patient has bilateral moyamoya disease with a right MCA occlusion and distal left ICA occlusion with a previous patent left EC-IC bypass.

**TABLE 1:** Healthy participant characteristics.

Age range years	All	Female	Male
18 to 35	13	6	7
36 to 60	9	1	8
61 to 85	2	0	2

### 2.2 The contrast agent: Transient hypoxia-induced dOHb protocol

**FIGURE 2:**
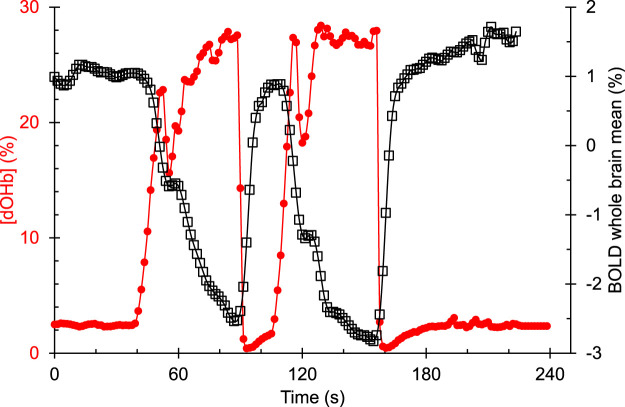
An example of the hypoxia-induced changes in [dOHb] (%) and the resulting whole brain average BOLD (%) signal response in a representative healthy control participant. [dOHb] was calculated from end tidal PO_2_ using the Hill equation describing the normal oxyhemoglobin *in-vivo* O_2_ dissociation curve (Balaban et al., 2013).

Changes in [dOHb] were achieved by controlling PetCO_2_ and PetO_2_ using sequential delivery of specific inspired gases with a computer-controlled gas blender (RespirAct™; Thornhill Medical Inc, Toronto, Canada) running a prospective targeting algorithm (Slessarev et al., 2007). The principles of operation of the RespirAct™ have been described elsewhere (Fisher et al., 2016). Participants breathed through a facemask sealed to the face with skin tape (Tegaderm, 3M, Saint Paul, MN, United States) to exclude all but system-supplied gas. The dOHb changes resulting from the programmed PetO_2_ stimulus pattern of 4-min and 20 s duration is shown in Figure 2. The pattern consisted of a 60 s normoxic baseline at PetO_2_ of 95 mmHg, a hypoxic step of PetO_2_ to 40 mmHg for 60 s, a return to normoxia for 20 s, a second hypoxic step for 60 s, followed by a return to normoxia for 60 s. After the completion of the PetO_2_ sequence, the participant returns to free breathing of room air. With this targeting approach, the end tidal values have been shown to be equal, within measurement error, to their respective arterial partial pressures (Ito et al., 2008; Fierstra et al., 2011).

### 2.3 MRI Scanning Protocol

A 3-Tesla scanner (HDx Signa platform, GE healthcare, Milwaukee, WI, USA) with an 8-channel head coil was used in these experiments. The protocol consisted of a high-resolution T1-weighted scan followed by one BOLD sequence scan. The high-resolution T1-weighted scan was acquired using a 3D spoiled gradient echo sequence with the following parameters: TI = 450 ms, TR 7.88 ms, TE = 3 ms, flip angle = 12°, voxel size = 0.859 × 0.859 × 1 mm, matrix size = 256 × 256, 146 slices, field of view = 24 × 24 cm, no interslice gap. The BOLD scan was acquired during normocapnic PetO_2_ manipulation using a T2*-weighted gradient echoplanar imaging sequence with the following parameters: TR = 1,500 ms, TE = 30 ms, flip angle = 73°, 29 slices voxel size = 3 mm isotropic voxels and matrix size = 64 × 64.

### 2.4 Data Analysis

The acquired BOLD images were volume registered, slice-time corrected and co-registered to the anatomical images using AFNI software (National Institutes of Health, Bethesda, Maryland) (Cox, 1996). Arterial oxygen saturation (SaO_2_) and [dOHb] were calculated from PetO_2_ and the oxyhemoglobin dissociation curve (Balaban et al., 2013) assuming a fixed [Hb] of 130 g/L and a pH of 7.4. Two methods of analysis were employed. First, a conventional analysis using an AIF chosen over the middle cerebral artery and a deconvolution-based model was used to calculate voxel-wise maps of rCBV and MTT. The rCBF was calculated as CBV/MTT and scaled by 25. This is described in greater detail elsewhere (Poublanc et al., 2021).

Second, a voxel-wise TFA of the BOLD data was analyzed using a custom program (LabVIEW, National Instruments, Texas). First, a zero-phase filter was applied to the BOLD vs. time data for all voxels to smooth the time course and reduce signal variations due to noise. The zero-phase filter applies an infinite impulse response recursive filter to the input signal such that the filtered signal has no phase distortion. Careful temporal alignment of the SO_2_ and BOLD data is required for the measurement of both Lag and Gain/Lag ratio. Lag is affected by both the contrast transit time and its time of arrival. Lag was minimised by using the whole brain population histogram as a guide, adjusting the alignment of SO_2_ and the whole brain mean BOLD to zero the minimum Lag. TFA calculated Gain, phase Lag, and Coherence as well as the Gain/Lag ratio for each voxel at the chosen frequency of 0.035 Hz. With this frequency the period is 28.57 s and phase Lag can be converted to time Lag (s) as period*phase lag (r/2π). SaO_2_ was taken as the measure of the contrast agent and used as the AIF.

**FIGURE 3:**
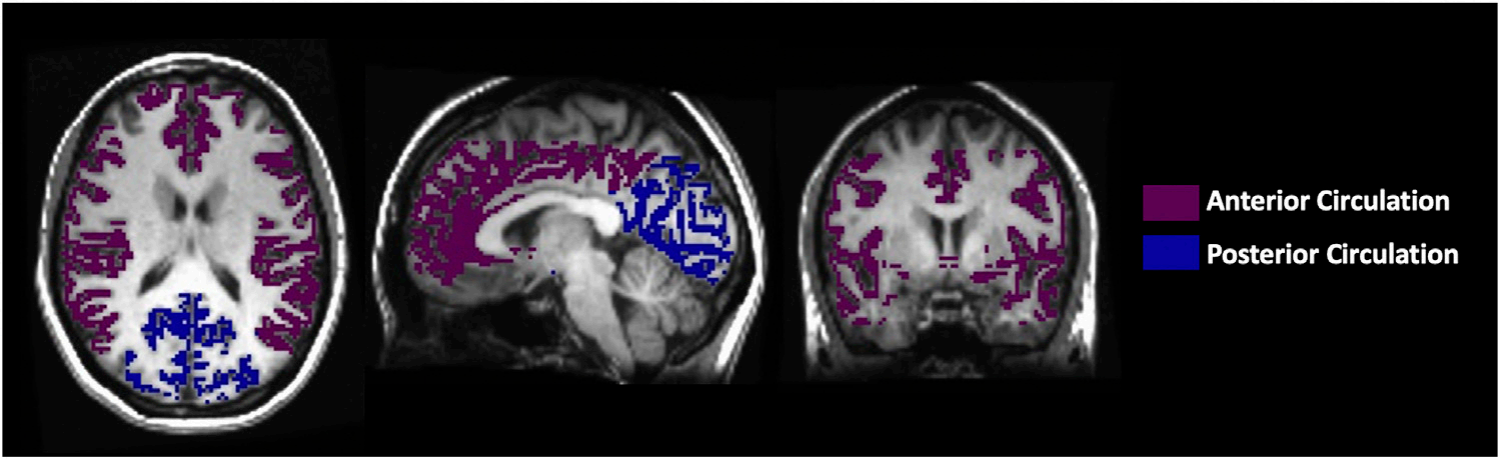
The axial, sagittal and coronal view of the manually delineated grey matter in the anterior and posterior circulation.

Maps of the perfusion metrics obtained from each analysis were transformed into Montreal Neurological Institute (MNI) space and overlayed onto their respective anatomical images. Analytical processing software, SPM8 (Wellcome Department of Imaging Neuroscience, Institute of Neurology, University College, London, UK), was used to segment the anatomical images (T1 weighted) into grey matter (GM) and white matter (WM). The vascular regions of interest (middle cerebral artery (MCA), posterior cerebral artery (PCA) and anterior cerebral artery (ACA)) were previously delineated manually on an anatomical MNI template. For this analysis, the supratentorial cortical grey matter MCA and ACA were combined as the anterior circulation mask and the supratentorial cortical grey matter PCA was used as the posterior circulation mask, as shown in Figure 3.

Average resting perfusion metrics using both TFA and conventional AIF analysis were calculated for specific regions for each participant in GM and WM and their ratios (GM/WM) using previously created vascular territory masks. To avoid susceptibility artifacts where the MRI signal is noisy, the slices ranging from mid to the top of the brain (slices 40–60) were selected to calculate perfusion metrics. Comparisons between the conventional AIF analysis and TFA for perfusion metrics were not possible for relative values (expressed in arbitrary units) and were therefore limited to only TFA lag vs. MTT (expressed in seconds). To assess the spatial discrimination of the two analyses, the regional GM/WM ratios were compared. The results from the grouped 24 healthy participants were compiled together to determine normative ranges for TFA (Gain, Lag, Coherence and Gain/Lag ratio), and for conventional AIF analysis (rCBV, MTT and rCBF). This was performed for each metric and analysis by calculating a voxel-by- voxel mean and standard deviation from the co-registered maps in standard space (Sobczyk et al., 2015; Sobczyk et al., 2021).

### 2.5 Statistical analysis

Comparisons were made using a two-way analysis of variance (ANOVA) with factors tissue region and type of analysis using a commercial statistical package (SigmaPlot, Systat Software, San Jose, California, USA). Both a Normality Test (Shapiro-Wilk) and Equal Variance Tests were part of the ANOVA, and correction for multiple comparisons were applied by an all pairwise multiple comparison procedure (Bonferroni method). The GM/WM ratios for MTT, rCBF and rCBV were compared between the types of analysis using one-way ANOVA. Significant difference in these tests was taken as *p* < 0.05.

## 3 Results

### 3.1 Group comparisons

**FIGURE 4:**
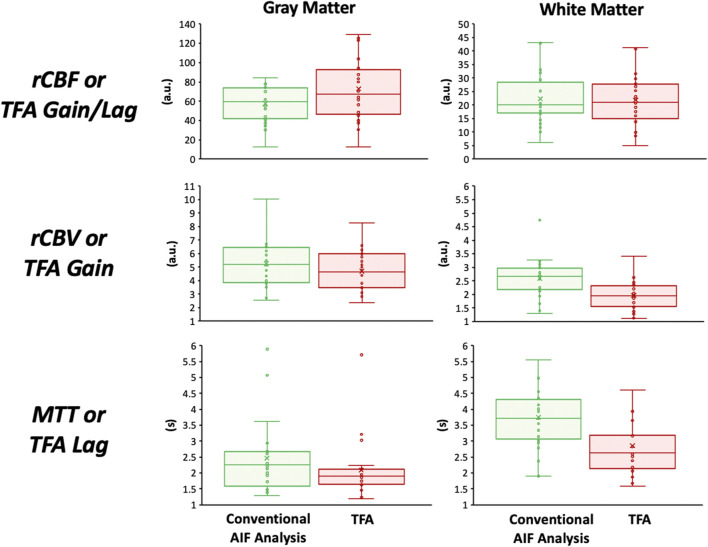
Boxplots showing the distribution of the perfusion metrics in the gray and white matter for the conventional AIF analysis (in green) and TFA (in red). The statistical summary comparing the two analysis techniques is found in Tables 2, 3.

**FIGURE 5:**
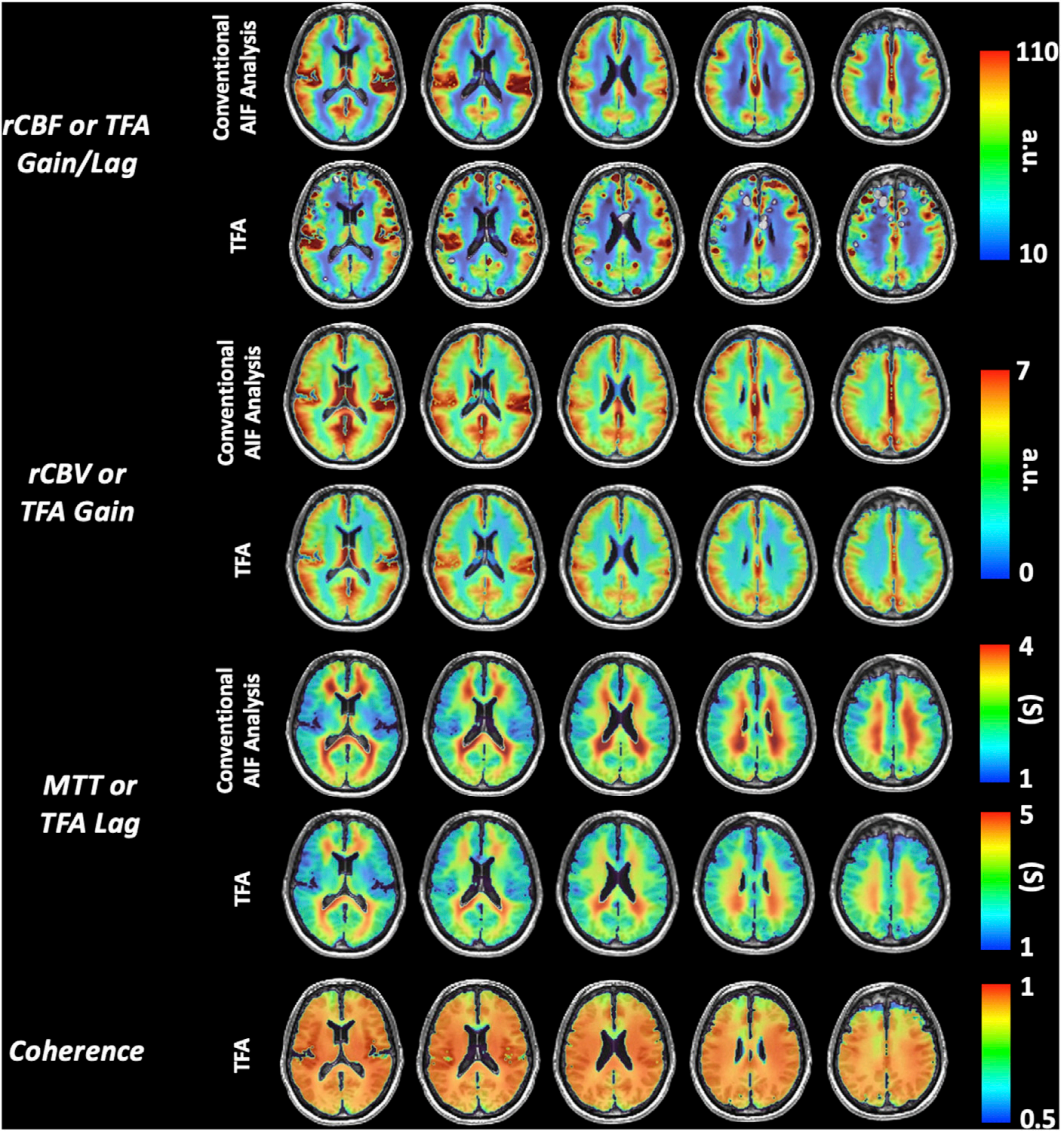
Representative axial slices of the grouped healthy participant group TFA metrics (Gain/Lag, Gain, Lag, and Coherence) and AIF metrics (rCBF, rCBV and MTT).

None of the subjects expressed distress during hypoxia and none terminated the procedure. Figure 4 presents the distribution of the data using boxplots. Figure 5 displays axial slices of the TFA and the conventional AIF analysis perfusion metrics for the grouped healthy participants. Note that colour scales minimum and maximum values were scaled to obtain the maximum colour contrast.

### 3.2 Statistical results

Table 2 summarises the comparison of TFA, and conventional AIF analysis metrics expressed as mean (SD) values for all the healthy participants. The Gain/rCBV and Gain/Lag/rCBF is relative and expressed in arbitrary units. Hence to be able to compare spatial discrimination between analysis methods, the GM/WM ratios were calculated. Table 3 summarises the comparison of GM/WM ratios between TFA, and conventional AIF analysis metrics expressed as mean (SD) values. The *p* values in Table 1 are from the two-way ANOVA with factors type of analysis and region. An All Pairwise Multiple Comparison Procedure (Bonferroni *t*-test) was used. The *p* values in Table 3 are from one-way ANOVA.

**TABLE 2:** A summary of the mean (SD) TFA lag vs. AIF MTT metrics for grouped healthy participants.

		Conventional AIF analysis			TFA			*p*-value lag vs. MTT
		rCBF	rCBV	MTT	Gain/Lag (“rCBF”)	Gain (“rCBV”)	Lag (“MTT”)	
GM		57.53 (18.33)	5.26 (1.65)	2.46 (1.11)	72.78 (32.08)	4.70 (1.50)	2.09 (0.94)	0.231
WM		22.30 (9.45)	2.60 (0.71)	3.74 (1.07)	21.80 (9.07)	1.96 (0.54)	2.86 (1.17)	0.006*
Anterior Circulation	GM	59.0 (18.44)	5.23 (1.62)	2.36 (1.09)	74.80 (33.48)	4.67 (1.45)	2.03 (0.95)	0.284
Posterior Circulation	GM	52.57 (19.09)	5.35 (1.75)	2.84 (1.24)	63.82 (27.52)	4.71 (1.63)	2.30 (0.90)	0.077

*p* values are from a 2-way ANOVA with asterisks (*) emphasising significant differences (*p* < 0.05).

**TABLE 3:** Whole brain supratentorial cortical GM/WM ratios for all metrics from both analyses.

Region	MTT	Lag	*p*-value
GM/WM	0.65 (0.17)	0.74 (0.13)	0.016*

	**rCBF**	**Gain/Lag**	*p* **-value**
	2.72 (0.65)	3.36 (0.70)	0.002*

	**rCBV**	**Gain**	*p* **-value**
	2.02 (0.27)	2.38 (0.32)	<0.001*

*p* values are from one-way ANOVAs with asterisks (*) marking significant differences (*p* < 0.05).

### 3.3 Examples of a healthy participant and patient

**FIGURE 6:**
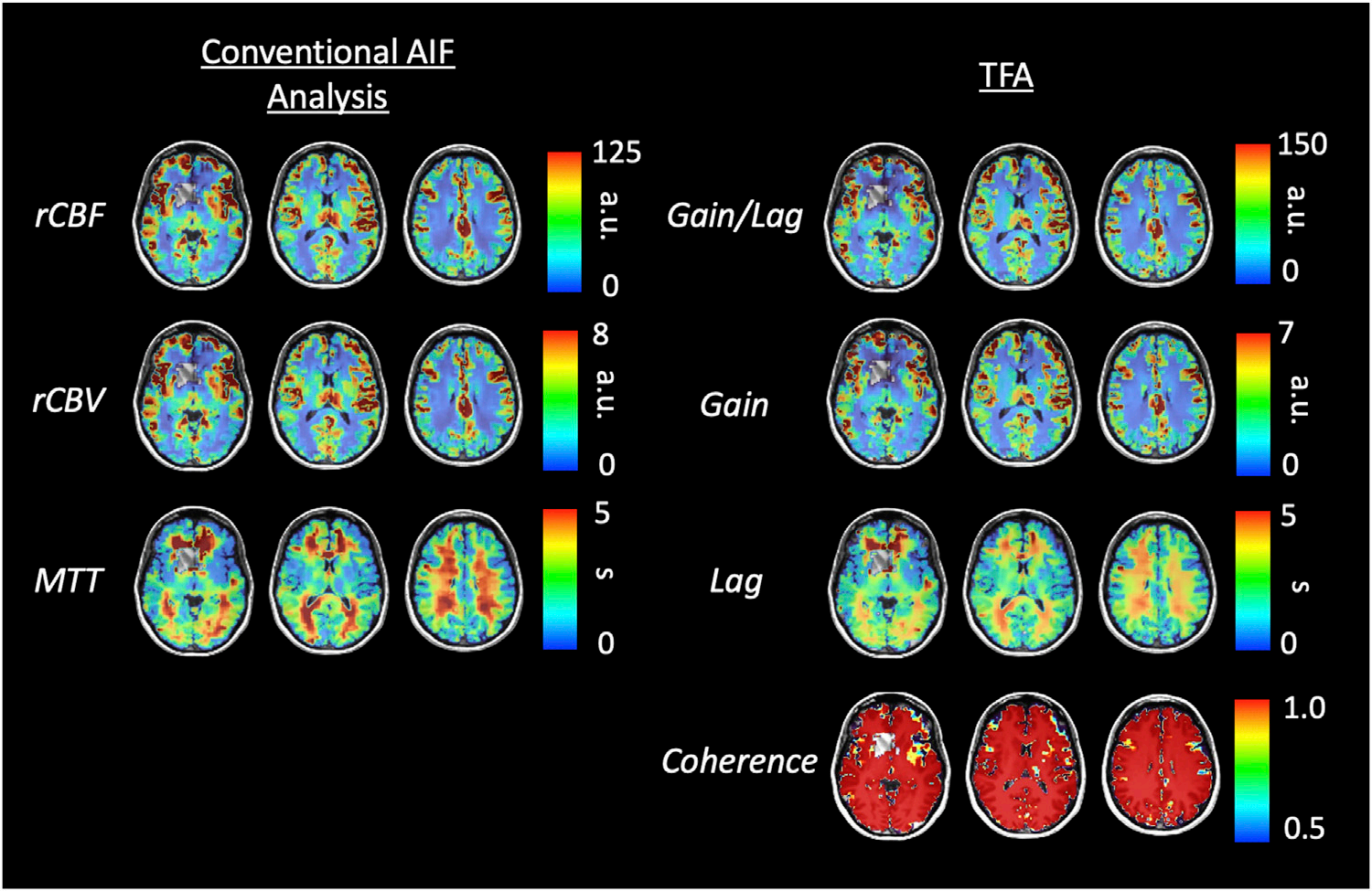
Axial slices of perfusion metrics for the conventional AIF and TFA analyses for a representative healthy control. Scales for rCBF, Gain/Lag, rCBV, and Gain are arbitrary units, and seconds for MTT and Lag.

**FIGURE 7:**
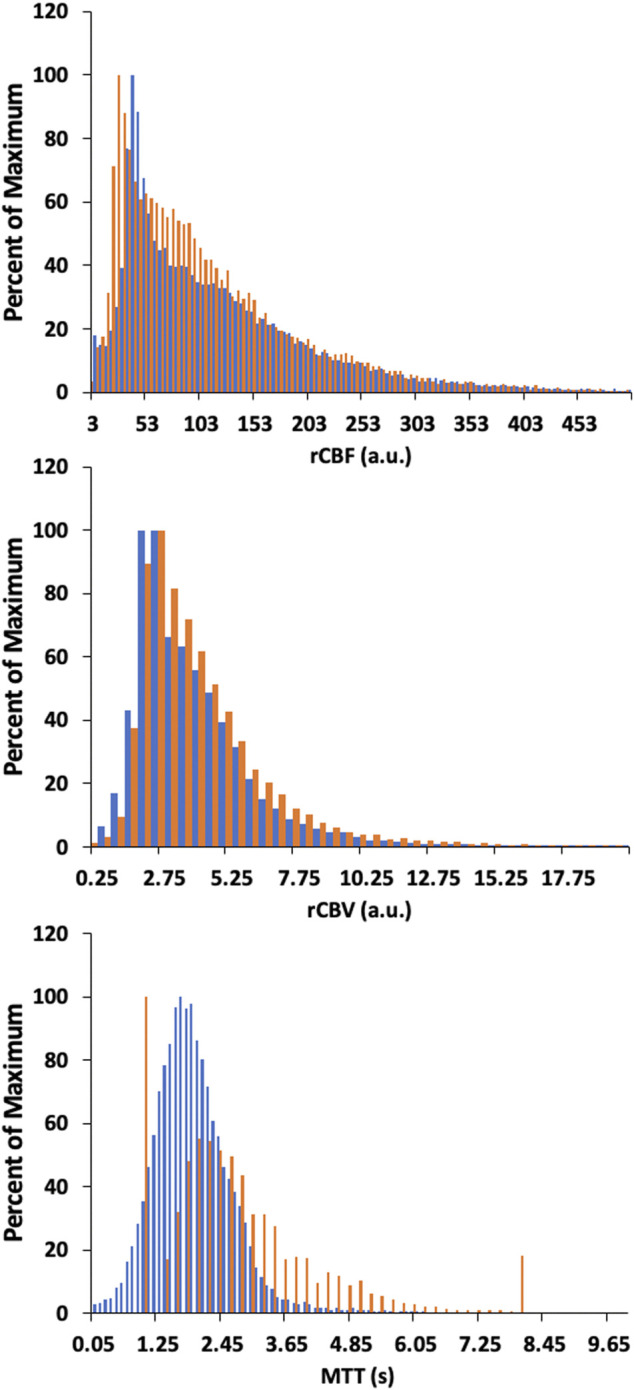
Histogram of whole brain averages for the conventional analysis (in orange) and TFA metrics (in blue) for a representative healthy control. Scales for rCBF, Gain/Lag, rCBV, and Gain are arbitrary units, and seconds for MTT and Lag.

**FIGURE 8:**
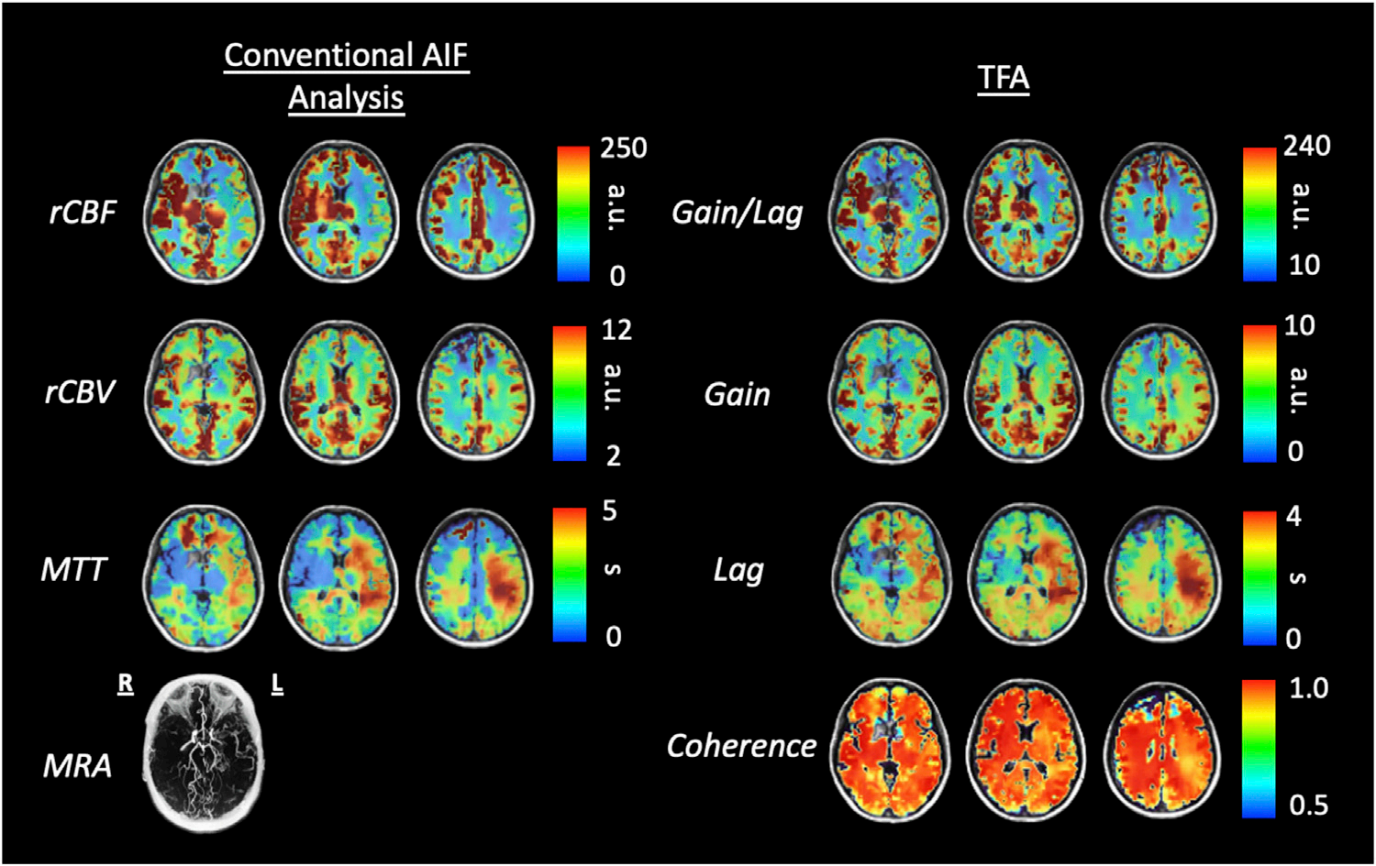
Axial slices of perfusion metrics for the conventional AIF analysis and TFA for a patient example with bilateral moyamoya disease with a right MCA occlusion and distal left ICA occlusion with a previous patent left EC-IC bypass. Note the decreased rCBF and Gain/Lag, the increased rCBV and Gain, and the prolonged MTT and Lag in all analyses reflect the vascular pathology of the patient. Scales for rCBF, Gain/Lag, rCBV and Gain are arbitrary units, seconds for MTT and Lag.

**FIGURE 9:**
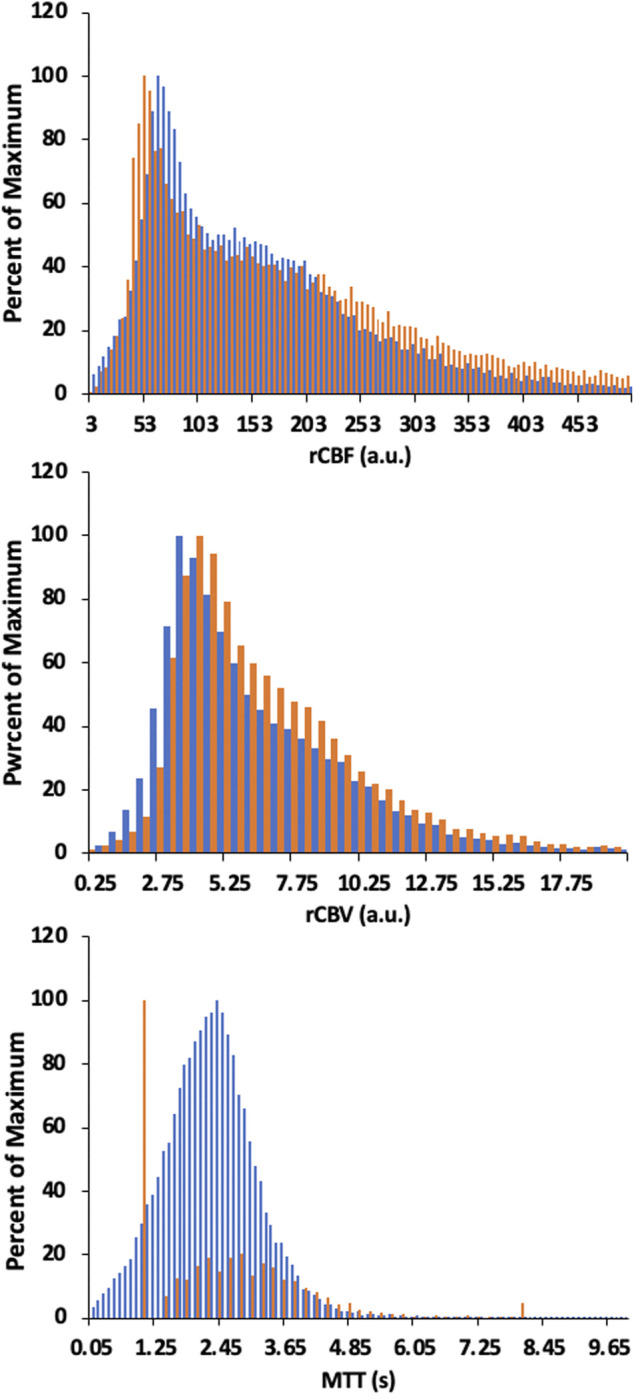
Histogram of whole brain averages for the conventional analysis (in orange) and TFA metrics (in blue) for a patient example with bilateral moyamoya disease with a right MCA occlusion and distal left ICA occlusion with a previous patent left EC-IC bypass. Scales for rCBF, Gain/Lag, rCBV, and Gain are arbitrary units, and seconds for MTT and Lag. Colour scales are adjusted to assist comparisons.

Figures 6, 8 display example perfusion metrics maps of a healthy individual and a selected patient. Figures 7, 9 display the histogram distribution of the perfusion metrics of a healthy individual and a selected patient.

## 4 Discussion

### 4.1 Main findings

We hypothesised that TFA metrics of Gain, Lag, and their ratio, Gain/Lag, correspond to conventional AIF resting perfusion metrics such as rCBV, MTT and rCBF, respectively. The main finding of this study is that TFA of the BOLD signal response to hypoxia induced dOHb provides perfusion metrics that are equivalent to those obtained with a conventional deconvolution-based analysis using an AIF. The grouped healthy participant maps of perfusion metrics for both types of analyses displayed high degree of similarity in relative magnitude and distribution of the perfusion metrics. The ranges of Lag and MTT values were similar with statistically significant but small differences only found in the whole brain WM. Ratios of GM/WM for each resting perfusion metric, calculated to assess their regional contrast, found Lag, Gain/Lag and Gain were significantly higher than MTT, rCBF and rCBV respectively, suggesting that TFA metrics provided a higher regional contrast.

We compared maps from a healthy individual to that of a patient with cerebrovascular pathology in order to determine whether TFA can identify cerebrovascular pathology seen with conventional AIF analysis. The comparison of perfusion values generated from conventional AIF analysis and TFA in the healthy individual show high congruence. Perhaps more importantly, there is also high spatial similarity between the two perfusion methods in magnitude and spatial distribution in the patient with right MCA occlusion and distal left ICA occlusion. This finding also suggests that TFA, like the AIF conventional analysis, can identify areas of reduced cerebrovascular health at rest.

Altogether these observations indicate that analysis of the BOLD response to THx-dOHb by TFA can provide an alternative to the conventional AIF analysis.

### 4.2 Detailed comparisons

Perfusion metrics rCBV and rCBF from the conventional AIF analysis and their TFA counterparts Gain, and Gain/Lag ratio are in arbitrary units so that direct numerical comparisons are not possible between analyses, or with published values of these metrics. Nevertheless, the grouped healthy participant maps for these metrics are spatially very similar to each other, and to other published maps of rCBV and rCBF, with regional differences that are in general agreement with the control group average maps presented by others (Grandin et al., 2005; Ibaraki et al., 2007; Newbould et al., 2007; Watabe et al., 2014; Asaduddin et al., 2019). Furthermore, the values in GM and WM fall within the range of those from computerized tomography studies (Chen et al., 2019).

Both MTT and Lag in grouped healthy participant maps depicted regional variations of about 0–5 s with whole brain means (SD) of 3.74 (1.07) and 2.86 (1.17) s in WM and 2.46 (1.11) and 2.09 (0.94) s in GM for MTT and Lag, respectively. These values are comparable to those found for DSC MRI of 3.0 (0.6) s in GM and 4.3 (0.7) s in WM by (Helenius et al., 2003), as well as those found using positron emission tomography and DSC MRI (Ibaraki et al., 2007; Wirestam et al., 2010). A range of 0–10 s were found using carpet plots to analyze transit times from low frequency oscillations in resting state fMRI (Fitzgerald et al., 2021) and from hypoxia-induced dOHb (Bhogal et al., 2022). The MTT and Lag metrics are also within the range of MTT metrics calculated previously using hypoxia-induced changes in [dOHb] as a susceptibility contrast agent; between 0 and 12 s (Vu et al., 2021) and between 0 and 8 s (Poublanc et al., 2021; Sayin et al., 2022a).

The maps of resting perfusion metrics and their corresponding histograms presented in Figures 6–9 provide two comparisons, one between a healthy control and a patient with cerebrovascular pathology, and the other between the two analysis methods TFA and conventional AIF analysis. Comparing the maps between the two methods demonstrate the apparent spatial similarity, with regional variations that match each other despite the different analytic approaches. The main differences between analyses with respect to the regional distributions of the metrics are discernible in the histograms, whose widths display the full variability of the metrics. As noted in Table 3 comparing the GM/WM ratios, the TFA discrimination between GM and WM is higher than the conventional AIF analysis.

We also note the very apparent differences between the perfusion maps of the healthy participant and that of the patient. These differences are also reflected in the histograms of the distribution of each metric. The maps of the patient example for both analyses indicate signs of the known left sided pathology with areas of increased MTT/Lag, increased rCBV/Gain and decreased rCBF/Gain/Lag ratio. We suggest that both analyses provide the clinically useful information.

### 4.3 Limitations

The voxel-wise application of TFA uses the changes in SO_2_ calculated from PetO_2_ measured at the lungs as the input signal and the measured BOLD changes in a voxel as the output signal. The voxel-wise TFA therefore assumes that the SO_2_ signal arrives at the voxel where BOLD is measured with the same changes as in pulmonary venous blood. Any dispersion is limited to passage through the left atrium and left ventricle, with the latter dispersion depending on the left ventricular ejection fraction.

One consideration in this study is the exposure of subjects to hypoxia. While SO_2_ and [dOHb] can be quickly returned to normal by increasing inspired oxygen to 100%, the speed of reduction of SO_2_ and [dOHb] by changing inspired PO_2_ is limited by minute ventilation, functional residual capacity, and oxygen consumption. The rate of washout of O_2_ from the functional residual capacity is limited by the lowest inspired oxygen concentration, which in the RespirAct™ is 4%, the functional residual capacity of the lungs, and minute ventilation. The two hypoxic exposures in the sequence were therefore extended to 60 s as the time to attaining PO_2_ of 40 mmHg is about 15–20 s, leaving at least 30 s of hypoxia baseline. Re-establishment of normoxia is usually evoked within one breath. This provides a step change in dOHb and can also act as a safety feature. An important caution is not to induce hypoxia in patients who are already hypoxic due to congenital heart disease, sickle cell disease, severe chronic obstructive and restrictive pulmonary disease, presence of lung atelectasis, pneumonia, asthma, and pulmonary shunting associated with COVID-19.

As Table 1 notes, the healthy participant group was small and varied in age and sex, so that the results cannot be differentiated by age or sex. Indeed, our purpose in recruiting was to sample a wide variety of individuals to gain a general sense of the range of perfusion metrics and their regional variation.

It is assumed that the brief hypoxic exposures do not cause any change in cerebral blood flow. This assumption is based partly on the observation that the vasculature does not begin to respond to hypoxia at resting PetCO_2_ until PetO_2_ is below 50 mmHg (Mardimae et al., 2012), and partly on the consideration that the vascular response time constant is too long (80 s) for the brief hypoxia to affect the flow (Poulin et al., 1996). We note that the conversion of PetO_2_ to SO_2_ assumed a fixed [Hb] of 130 g/L and a pH = 7.4 and suggest that if actual measures are available, adjustments to the calculation of perfusion metrics be applied for each individual.

## 5 Conclusion

In this study THx-dOHb was used to produce a rapid variation in [dOHb], which induced sufficient BOLD signal changes to enable the calculation of resting perfusion metrics using TFA. We hypothesised that perfusion metrics derived from TFA analysis would be congruent with those derived from standard DSC processing using deconvolution of the AIF with the tissue response function such that TFA Gain would be analogous to rCBV, TFA Lag to MTT and the ratio Gain/Lag to rCBF. The truth of this hypothesis was first verified by showing high congruence between perfusion maps generated by both analytical methods in a healthy group of participants. A second confirmation was obtained by showing that TFA was able to discriminate between healthy and diseased tissue in a patient with a right MCA occlusion and distal left ICA occlusion with a previous patent left EC-IC bypass in a manner that was also highly consistent with the conventional analysis. We conclude that TFA of the BOLD changes resulting from THx-dOHb can be used to provide an alternative analysis method of determining resting perfusion metrics in individuals, which eliminates the requirement of an AIF selection and complex deconvolution calculations based on an assumed kinetic model. Furthermore, TFA has the potential to be applied to perfusion analysis for DSC imaging using GBCAs.

## Data Availability

The original contributions presented in the study are included in the article/Supplementary Material, further inquiries can be directed to the corresponding author.
